# Supplementary material for: Hepatic transcriptome analysis identifies genes, polymorphisms and pathways involved in the fatty acids metabolism in sheep
Source: PLoS One. 2021 Dec 23;16(12):e0260514. doi: 10.1371/journal.pone.0260514 (PMC8699643; doi:10.1371/journal.pone.0260514)
Supplement: S5 Table — (DOCX) [file pone.0260514.s005.docx]

| Polymorphism position | Chr | Number of sheep | Genotype frequency | | |  | Allele frequency | | Chi-square test |
| --- | --- | --- | --- | --- | --- | --- | --- | --- | --- |
|  |  |  | CC | CT | TT |  | C | T | χ^2^ |
| APOA5 g.37284 C>T | 15 | 100 | 0.56 (56) | 0.38 (38) | 0.06 (6) |  | 0.75 | 0.25 | 0.018 |
|  |  |  | CC | CT | TT |  | C | T | χ^2^ |
| CFHR5 g.1011 C>T | 12 | 100 | 0.38 (38) | 0.49 (49) | 0.13 (13) |  | 0.63 | 0.37 | 0.206 |
|  |  |  | AA | AG | GG |  | A | G | χ^2^ |
| TGFBR2 g.5112179 A>G | 19 | 100 | 0.82 (82) | 0.15 (15) | 0.03 (3) |  | 0.90 | 0.10 | 4.077 |
|  |  |  | AA | AC | CC |  | A | C | χ^2^ |
| LEPR g.40761672 A>C | 1 | 100 | 0.64 (64) | 0.32 (32) | 0.04 (4) |  | 0.80 | 0.20 | 0.000 |

Supplementary Table S5 Genotype, allele frequencies, and chi-square test of selected SNPs validated using RFLP
