# Supplementary material for: Hepatic transcriptome analysis identifies genes, polymorphisms and pathways involved in the fatty acids metabolism in sheep
Source: PLoS One. 2021 Dec 23;16(12):e0260514. doi: 10.1371/journal.pone.0260514 (PMC8699643; doi:10.1371/journal.pone.0260514)
Supplement: Attachment — Submitted filename: Reviewer response letter.docx [file pone.0260514.s006.docx]

**Response to Editor**

**Comment**: PONE-D-21-19113
Hepatic transcriptome analysis identifies genes, polymorphisms and pathways involved in the fatty acids metabolism in sheep
PLOS ONE

Dear Dr. Asep Gunawan,
Thank you for submitting your manuscript to PLOS ONE. After careful consideration, we feel that it has merit but does not fully meet PLOS ONE’s publication criteria as it currently stands. Therefore, we invite you to submit a revised version of the manuscript that addresses the points raised during the review process.

Please submit your revised manuscript by Sep 17 2021 11:59PM. If you will need more time than this to complete your revisions, please reply to this message or contact the journal office at plosone@plos.org. When you're ready to submit your revision, log on to https://www.editorialmanager.com/pone/ and select the 'Submissions Needing Revision' folder to locate your manuscript file.

Please include the following items when submitting your revised manuscript:

- A rebuttal letter that responds to each point raised by the academic editor and reviewer(s). You should upload this letter as a separate file labelled 'Response to Reviewers'.
- A marked-up copy of your manuscript that highlights changes made to the original version. You should upload this as a separate file labelled 'Revised Manuscript with Track Changes'.
- An unmarked version of your revised paper without tracked changes. You should upload this as a separate file labelled 'Manuscript'.

If you would like to make changes to your financial disclosure, please include your updated statement in your cover letter. Guidelines for resubmitting your figure files are available below the reviewer comments at the end of this letter.

If applicable, we recommend that you deposit your laboratory protocols in protocols.io to enhance the reproducibility of your results. Protocols.io assigns your protocol its own identifier (DOI) so that it can be cited independently in the future. For instructions see: <http://journals.plos.org/plosone/s/submission-guidelines#loc-laboratory-protocols>. Additionally, PLOS ONE offers an option for publishing peer-reviewed Lab Protocol articles, which describe protocols hosted on protocols.io. Read more information on sharing protocols at <https://plos.org/protocols?utm_medium=editorial-email&utm_source=authorletters&utm_campaign=protocols>.

We look forward to receiving your revised manuscript.
Kind regards,
Islam Hamim, PhD
Academic Editor
PLOS ONE

**Response:** We would like to thank the editor for marking our manuscript ‘interested’ and for detailed guidance to make the manuscript publishable. We would like to take this opportunity to thank the reviewers for their valuable time and comments. We do believe that these comments improve the manuscript significantly. We have responded all the comments from the reviewers point by point.

**Response to the journal office and /or Editor**

**Comment:** Journal Requirements:

When submitting your revision, we need you to address these additional requirements.

1. Please ensure that your manuscript meets PLOS ONE's style requirements, including those for file naming. The PLOS ONE style templates can be found at 
<https://journals.plos.org/plosone/s/file?id=wjVg/PLOSOne_formatting_sample_main_body.pdf> and 
<https://journals.plos.org/plosone/s/file?id=ba62/PLOSOne_formatting_sample_title_authors_affiliations.pdf>

2. In your Methods section, please provide the name of the slaughterhouse where the animals were sacrificed

**Response**: Thank you for this point. We have added the name of slaughterhouse in the revised manuscript. Please see the Method ‘PT Pramana Pangan Utama’ which is located in IPB University (line no. 447-448). The file naming and necessary formatting have been done. Please see the revised manuscript with track changes.

**Comment:** 3. We noticed you have some minor occurrence of overlapping text with the following previous publication(s), which needs to be addressed:

- <https://www.sciencedirect.com/science/article/pii/S2452014419300123?via%3Dihub>
In your revision ensure you cite all your sources (including your own works), and quote or rephrase any duplicated text outside the methods section. Further consideration is dependent on these concerns being addressed.

**Response:** We appreciated the suggestions. The above-mentioned manuscript is published by our group (Gunawana A, Listyarinia K, Furqona A, Jakaria, Sumantri C, Akter SH and Uddin MJ. *RNA deep sequencing reveals novel transcripts and pathways involved in the unsaturated fatty acid metabolism in chicken*. Gene Reports, 15: 100370. 2019.) which was a similar study performed in chicken. In this manuscript we performed more vigorous analysis, as well as association studies for 4 SNPs in 4 different candidate genes and expression validation of 8 candidate genes using qRT-PCR. We ensure you that there is no overlapping. Note, the manuscript has been cited in the revised manuscript.

**Comment:** 4. We note that you have stated that you will provide repository information for your data at acceptance. Should your manuscript be accepted for publication, we will hold it until you provide the relevant accession numbers or DOIs necessary to access your data. If you wish to make changes to your Data Availability statement, please describe these changes in your cover letter and we will update your Data Availability statement to reflect the information you provide.

**Response:** Thanks. Please change the Data Availability’ statement. The data has been submitted to NCBI (Accession: PRJNA764003, ID: 764003). We apologies.

**Comment:** 5. Thank you for stating the following in the Acknowledgments Section of your manuscript: "This work was supported by a project World Class Research (WCR) Number: 077/SP2H/LT/DRPM/2021 from the Ministry of Agriculture of the Republic of Indonesia."

We note that you have provided funding information that is not currently declared in your Funding Statement. However, funding information should not appear in the Acknowledgments section or other areas of your manuscript. We will only publish funding information present in the Funding Statement section of the online submission form. 
Please remove any funding-related text from the manuscript and let us know how you would like to update your Funding Statement. Currently, your Funding Statement reads as follows:  "This work was supported by a project World Class Research (WCR) Number: 077/SP2H/LT/DRPM/2021 from the Ministry of Agriculture of the Republic of Indonesia."

Please include your amended statements within your cover letter; we will change the online submission form on your behalf.

**Response:** Please update the funding details in the ‘Funding Statement section of the online submission form’ on Author’s behalf. We appreciated your cooperation.

**Comment:** 6. We note that you have included the phrase “data not shown” in your manuscript. Unfortunately, this does not meet our data sharing requirements. PLOS does not permit references to inaccessible data. We require that authors provide all relevant data within the paper, Supporting Information files, or in an acceptable, public repository. Please add a citation to support this phrase or upload the data that corresponds with these findings to a stable repository (such as Figshare or Dryad) and provide and URLs, DOIs, or accession numbers that may be used to access these data. Or, if the data are not a core part of the research being presented in your study, we ask that you remove the phrase that refers to these data.

**Response:** The phrase ‘data not shown’ has been removed, please see the revised manuscript with track changes.

**Comment:** Additional Editor Comments (if provided):

This is an interesting study, but the manuscript needs to be rewritten to reflect the reviewers' suggestions. I recommend that the authors make the major revisions that have been suggested by reviewers.
**Response:** Thanks for the encouraging comment. We have edited the manuscript following reviewer’s comments. Please see the revised manuscript with track changes.

**Response to reviewers’ comments**

 Comments to the Author
1. Is the manuscript technically sound, and do the data support the conclusions?
Reviewer #1: Partly

Reviewer #2: Yes

Reviewer #3: Partly

2. Has the statistical analysis been performed appropriately and rigorously?

Reviewer #1: N/A

Reviewer #2: Yes

Reviewer #3: No

3. Have the authors made all data underlying the findings in their manuscript fully available?

Reviewer #1: No

Reviewer #2: No

Reviewer #3: Yes

4. Is the manuscript presented in an intelligible fashion and written in standard English?
Reviewer #1: No

Reviewer #2: No

Reviewer #3: Yes

5. Review Comments to the Author

Please use the space provided to explain your answers to the questions above. You may also include additional comments for the author, including concerns about dual publication, research ethics, or publication ethics. (Please upload your review as an attachment if it exceeds 20,000 characters)

**Response:** Thanks for the comments. We have edited the manuscript following reviewer’s comments. Please see the response below as well as the revised manuscript with track changes.

**Response to reviewer #1**

**Comment:** Reviewer #1: The study by Asep Gunawan et al. aims to elucidate genes and pathways involved in fatty acid metabolism using RNA sequencing technology to reveal differentially expressed genes in the liver tissues from sheep with high and low unsaturated fatty acid. In addition, the authors profile gene expression of putative new candidate genes for high and low unsaturated fatty acid and analyze its association with the phenotype under study. The paper has some merit in that it reports the association of DNA variants with high/low unsaturated fatty acid, following what is common practice in the study of candidate genes. Unfortunately, I have a number of concerns below detailed in no specific order.
**Response:** We would like to thank the reviewer #1 for his/her time and valuable comments. We have responded all the comments point by point.

**Comment:** 1. The use of language makes the paper hard to understand. The manuscript needs thorough language editing; authors must provide certified proof of English language editing.
**Response:** We have revised the manuscript for language. An English native speaker (expert in Animal Science, Murdoch University, Australia) has volunteered in this regard.

**Comment:** 2. Abstract- #Line40, high USFA, and low SFA; and/or higher or lower USFA? in #LINE 43-44. please, choose the right word to describe the traits.
**Response:** Thanks for pointing this out. Since these are comparative traits so we have decided to use ‘higher’ and ‘lower’ rather than high and low USFA. Please see the revised manuscript with track changes.

**Comment:** 3. I am an animal geneticist and functional genomics analyst, so I think that the Introduction section does a poor job.
**Response:** Apologies for unwilling mistake. The introduction has been modified and improved. Please see the changes in the revised manuscript with track change.

**Comment:** 4. Table 1 is not clear….#line920 (^ab^Mean value with different superscript letters in the same row differ significantly at P<0.05 ) ab-superscript is missing in Table 1, or is there no significant difference between the traits measured?
**Response:** Thank you for pointing this out. We apologize for the unwilling mistake. The superscript has been added in the revised Table 1.

**Comment:** 5. #Line 43-44, and #line 136-137-. These sentences are contracting and not clear. Authors need to be more specific in the choice of words used to describe high and low fatty acids.
**Response:** Apologies for creating confusion unwilling. The sentences have been revised in the revised manuscript (line no: 140-141).

**Comment:** 6. #Table 3, I can’t see the list of up-and down-regulated genes as described by the authors. I would suggest an additional column representing up and down-regulated genes between sheep with high and low unsaturated fatty acids.
**Response:** Thank you for this point. The table is getting large, so we have added a superscript (**^¥^**) and foot note in the table 3 to indicate up and down regulated gene. Please see the revised manuscript (at the end of Table 3).

**Comment:** 7. More than six pages of discussion seem too much, despite the authors didn’t discuss the causative mutations involved in regulating high and low unsaturated fatty acids in sheep.
**Response:** The ‘Discussion’ has been modified and improved. Please see the changes in the revised manuscript with track change. Please note that this study includes vigorous sequence analysis, as well as association studies for 4 novel SNPs in 4 different candidate genes, and expression validation of 8 candidate genes using qRT-PCR. We have our best to explain and discussion the important findings.

**Comment:** 8. The sequencing datasets from this study cannot be accessed. The authors should provide the accession number.
**Response:** The data has been submitted to NCBI (Accession: PRJNA764003, ID: 764003). We apologies.

**Response to reviewer #2**

**Comment:** Reviewer #2: I think this experiment is very meaningful. However, there are some major problems with the manuscript.
Figure 3, 4, 5, 6 not found in the manuscript, and Figures are not clear. Authors are encouraged to resubmit.

**Response:** Thank you so much for your time and encouraging comment. We have submitted all the figures separately along with manuscript according to the Plos One’s Author instructions. We have double checked the figures quality. We apologies for the issue.

**Response to reviewer #3**

**Comment:** Reviewer #3: 1. Sheep having USFA >45.59 % μg/g and <25.84 % μg/g was considered as high-USFA (HUSFA) and low-USFA (LUSFA) group, respectively. But in the table 1, the mean USFA of HUSFA and LUSFA were 25.84% and 45.59%, respectively. So, the meaning of two sentences does not coincide.
**Response:** Thank you so much for your time and encouraging comment. We apologies for the unwilling mistake. The statement has been revised and corrected. Please see the revised manuscript (line no: 468-470): ‘Sheep having USFA ≥45.59 % μg/g and ≤25.84 % μg/g was considered as higher-USFA (HUSFA) and lower-USFA (LUSFA) group, respectively’.

**Comment:** 2. A total 100 sheep were slaughtered, and the blood samples were taken for DNA extraction for validation of SNP and association study. Weather the 100 sheep were the same as the 100 sheep for FA analysis? The authors did not make clear.
**Response:** We appreciate for the comments. The same 100 sheep were used for both association study and fatty acid analysis. The statement has been made clear in the revised manuscript with track change (please see line no. 446-447).

**Comment**: 3. The average expression values of GAPDH and β-Actin was used to normalize the gene expression value. Why select these two genes? According to experiment test or other’s reports? The reason or criteria to select GAPDH and β-Actin should write clearly.

**Response**: We appreciated for this point. We have selected these two housekeeping genes based on our previous report conducted with muscle tissue in sheep (Gunawan et al, 2018) published in Gene, 676: 86-94.

Reference

**Gunawan**, A., Jakaria, K Listyarini, A Furqon, C Sumantri, SH Akter, MJ Uddin. 2018. Transcriptome signature of liver tissue with divergent mutton odor and flavour using RNA deep sequencing. *Gene.* 676: 86-94.
