# Supplementary material for: Hepatic transcriptome analysis identifies genes, polymorphisms and pathways involved in the fatty acids metabolism in sheep
Source: PLoS One. 2021 Dec 23;16(12):e0260514. doi: 10.1371/journal.pone.0260514 (PMC8699643; doi:10.1371/journal.pone.0260514)
Supplement: Attachment — Submitted filename: Response to Reviewers.docx [file pone.0260514.s007.docx]

**Response to academic editor**

**Comment**: PONE-D-21-19113R1

Hepatic transcriptome analysis identifies genes, polymorphisms and pathways involved in the fatty acids metabolism in sheep

PLOS ONE
Dear Dr. Gunawan,
Thank you for submitting your manuscript to PLOS ONE. After careful consideration, we feel that it has merit but does not fully meet PLOS ONE’s publication criteria as it currently stands. Therefore, we invite you to submit a revised version of the manuscript that addresses the points raised during the review process.
Please submit your revised manuscript by Nov 22 2021 11:59PM
We look forward to receiving your revised manuscript.
Kind regards,
Martina Zappaterra
Academic Editor
PLOS ONE
**Response**: We would like to thanks the editor and reviewers for marking the manuscript as ‘*minor-revision*’ and for their valuable time and comments. We do believe that these comments have improved the quality of the manuscript. English has been double checked with native English reader expert in animal science. All changes and corrections could be found in the revised manuscript with track change.

**Comment**: Please review your reference list to ensure that it is complete and correct. If you have cited papers that have been retracted, please include the rationale for doing so in the manuscript text, or remove these references and replace them with relevant current references. Any changes to the reference list should be mentioned in the rebuttal letter that accompanies your revised manuscript. If you need to cite a retracted article, indicate the article’s retracted status in the References list and also include a citation and full reference for the retraction notice.
**Response**: Recent and appropriate references have been cited in this manuscript. We were careful to give credit to the original articles. All the references have been double checked both in the text and in the reference section. The references style has been corrected following journal style and according to author instructions. All changes and corrections could be found in the revised manuscript with track change.

**Comment**: Additional Editor Comments (if provided):
Reviewers have evaluated positively the manuscript. However, in order to avoid repetition and grammatical errors, authors are encouraged to have their manuscript professionally edited in English.
**Response**: English has been double checked with native English speaker expert in animal science. All changes and corrections could be found in the revised manuscript with track change.

[Note: HTML markup is below. Please do not edit.]

**Response to Reviewers' comments**

Reviewer's Responses to Questions

**Comment**: **Comments to the Author**
1. If the authors have adequately addressed your comments raised in a previous round of review and you feel that this manuscript is now acceptable for publication, you may indicate that here to bypass the “Comments to the Author” section, enter your conflict of interest statement in the “Confidential to Editor” section, and submit your "Accept" recommendation.

Reviewer #1: (No Response)

Reviewer #3: All comments have been addressed

**Comment**: 2. Is the manuscript technically sound, and do the data support the conclusions?
The manuscript must describe a technically sound piece of scientific research with data that supports the conclusions. Experiments must have been conducted rigorously, with appropriate controls, replication, and sample sizes. The conclusions must be drawn appropriately based on the data presented.

Reviewer #1: Yes

Reviewer #3: Yes

**Comment**: 3. Has the statistical analysis been performed appropriately and rigorously?

Reviewer #1: N/A

Reviewer #3: Yes

**Comment**: 4. Have the authors made all data underlying the findings in their manuscript fully available?
The [PLOS Data policy](http://www.plosone.org/static/policies.action#sharing) requires authors to make all data underlying the findings described in their manuscript fully available without restriction, with rare exception (please refer to the Data Availability Statement in the manuscript PDF file). The data should be provided as part of the manuscript or its supporting information, or deposited to a public repository. For example, in addition to summary statistics, the data points behind means, medians and variance measures should be available. If there are restrictions on publicly sharing data—e.g. participant privacy or use of data from a third party—those must be specified.

Reviewer #1: Yes

Reviewer #3: Yes

**Comment**: 5. Is the manuscript presented in an intelligible fashion and written in standard English?
PLOS ONE does not copyedit accepted manuscripts, so the language in submitted articles must be clear, correct, and unambiguous. Any typographical or grammatical errors should be corrected at revision, so please note any specific errors here.

Reviewer #1: (No Response)

Reviewer #3: Yes

**Comment**: 6. Review Comments to the Author
Please use the space provided to explain your answers to the questions above. You may also include additional comments for the author, including concerns about dual publication, research ethics, or publication ethics. (Please upload your review as an attachment if it exceeds 20,000 characters)

Reviewer #1: Dear Authors, I appreciate your efforts to improve your manuscript and your decision to publish it in PLoS One. However, in order to avoid repetition and grammatical errors, authors must have their manuscript professionally edited in English.
These are just a few of the grammatical errors discovered....:
In L43 delete “a” . From sheep (n=100)....(Plural form)
L109 "analysis" In addition, gene polymorphism and association analyses......(Plural form)....

Reviewer #3: (No Response)

**Response**: We thanks the reviewer for pointing this out. We apologies for unwilling grammatical errors. English has been double checked with native English speaker expert in animal science. All changes and corrections could be found in the revised manuscript with track change.

**Comment**: 7. PLOS authors have the option to publish the peer review history of their article ([what does this mean?](https://journals.plos.org/plosone/s/editorial-and-peer-review-process#loc-peer-review-history)). If published, this will include your full peer review and any attached files.
If you choose “no”, your identity will remain anonymous but your review may still be made public.
**Do you want your identity to be public for this peer review?** For information about this choice, including consent withdrawal, please see our [Privacy Policy](https://www.plos.org/privacy-policy).

Reviewer #1: No

Reviewer #3: No

**Response**: We would like to thank the editor and reviewers for their valuable time and comments. We do believe that these comments have improved the quality of the manuscript. The revision has been submitted according to the journal instructions.
